# Long-term efficacy and risk factors for stent occlusion in portal vein stent placement: a multi-institutional retrospective study

**DOI:** 10.1186/s42155-022-00307-0

**Published:** 2022-06-16

**Authors:** Hirotsugu Nakai, Hironori Shimizu, Takanori Taniguchi, Seiya Kawahara, Toshihide Yamaoka, Naoya Sasaki, Hiroyoshi Isoda, Yuji Nakamoto

**Affiliations:** 1 Department of Radiology, Tenri Hospital, 200 Mishima-cho, Tenri, Nara 632-8552 Japan; 2 Department of Diagnostic Imaging and Nuclear Medicine, Kyoto University Graduate School of Medicine, 54 Kawahara-cho, Shogoin, Sakyo-ku, Kyoto, 606-8507 Japan; 3 Department of Radiology, Otsu Red Cross Hospital, Nagara 1-1-35, Otsu, Shiga 520-0046 Japan; 4 Department of Diagnostic Imaging and Interventional Radiology, Kyoto Katsura Hospital, 17 Yamada-Hirao, Nishikyo, Kyoto, 615-8256 Japan; 5 Department of Surgery, Kyoto Katsura Hospital, 17 Yamada-Hirao, Nishikyo, Kyoto, 615-8256 Japan; 6 Preemptive Medicine and Lifestyle Disease Research Center, Kyoto University Hospital, 53 Kawahara-cho, Shogoin, Sakyo-ku, Kyoto, 606-8507 Japan

**Keywords:** Hypertension, Portal, Portal vein, Stents, Gastrointestinal hemorrhage, Ascites

## Abstract

**Background:**

Surgical treatment for PV (portal vein) stenosis/occlusion can pose a fatal risk of massive bleeding from severe adhesions and collateral vessel formation. PV stents placement is a minimally invasive and effective procedure for PV stenosis/occlusion, but PV stents sometimes occlude. The relationship between post-stent PV hemodynamics and stent occlusion has not been thoroughly investigated. Certain precautions during PV stent placement may reduce the risk of stent occlusion. This study aimed to evaluate long-term outcomes of PV stent patency and investigate factors including PV hemodynamics associated with stent occlusion.

**Materials and methods:**

Thirty-four consecutive patients with PV stenosis/occlusion who underwent PV stent placement in four institutions between December 2006 and February 2021 were retrospectively examined. The primary study endpoints were technical success, clinical success, and cumulative stent patency rate. The secondary endpoints were risk factors of stent occlusion. A univariable Cox proportional hazards model with sixteen variables was used to determine predictors of stent occlusion. Factors with *p*-value ≤ 0.1 in univariable analysis were included in the multivariable analysis. Alpha was set at 0.05.

**Results:**

Technical and clinical success rates were 88.2% and 79.4%, respectively. Six patients (17.7%) experienced stent occlusion. The cumulative stent patency rate at six months, one year, and three years was 79.1%, 79.1%, and 65.9%, respectively. In the univariate analysis, the variables with *p*-value ≤ 0.1 were lesion length > 4 cm, hepatofugal collateral vein visualization after stent placement, and residual stenosis > 30% after stent placement. In the multivariate analysis, residual stenosis > 30% after stent placement was significantly associated with stent occlusion (hazard ratio, 10.80; 95% confidence interval, 1.08–108.44; *p* = 0.04).

**Conclusion:**

PV stent placement was technically feasible and effective in improving portal hypertension. However, stent occlusion was not uncommon. Residual stenosis > 30% after stent placement was significantly associated with stent occlusion. We should pay attention to correctly assess the range of stenosis and release the stenosis as much as possible.

## Background

Portal vein (PV) stenosis/occlusion can occur because of reduced portal flow velocity, tumor encasement, or as postoperative complications (Kumar et al. 2015). The reported incidence of PV stenosis/occlusion is 2.4% after pancreaticoduodenectomy (Hiyoshi et al. 2015) and < 3% after liver transplantation (Woo et al. 2007; Settmacher et al. 2000). It can cause liver damage, gastrointestinal bleeding, and refractory ascites. Previous studies have shown that PV stent placement is a relatively safe and effective treatment that improves hepatic blood flow and portal hypertension (Yamakado et al. 2001a; Hasegawa et al. 2015; Lee et al. 2021). However, PV stents sometimes occlude, which has been associated with severe hepatic dysfunction (Yamakado et al. 2001a), pancreatic juice leakage (Lee et al. 2021), and splenic vein involvement (Yamakado et al. 2001b). As far as we know, a multi-institutional study about PV stent placement has not been conducted, and more generalizable results of the long-term efficacy data regarding PV stent placement is warranted. In addition, the relationship between post-stent PV hemodynamics and stent occlusion has not been thoroughly investigated. Presence of residual collateral veins after stent placement or residual PV stenosis may be associated with stent occlusion. If this is the case, certain precautions during stent placement may reduce the risk of stent occlusion. This study aimed to evaluate long-term outcomes of PV stent patency and investigate factors associated with stent occlusion.

## Material and methods

### Patients

Thirty-four consecutive patients aged 21 years or older who underwent PV stent placement in four institutions between December 2006 and February 2021 were enrolled. Study approval was obtained from the institutional review board of each participating hospital. The informed consent requirement was waived.

### Stent placement

Stent placement was intended to alleviate or prevent portal hypertension-related symptoms (gastrointestinal bleeding, encephalopathy, refractory ascites), mitigate liver dysfunction, or facilitate chemotherapy. With informed consent, PV stents were placed by board-certified interventional radiologists via ultrasonography (US)-guided percutaneous transhepatic approach or open trans-ileocecal approach. The approach was determined on an individual basis according to general condition, and presence of ascites. In the percutaneous transhepatic approach, PV segment V was preferentially chosen, while segment III was accessed for patients who had undergone right hepatectomy. After PV puncture with a 21-gauge needle (Hanako Medical, Saitama, Japan) under local anesthesia, a 0.018-inch guidewire was first inserted and then exchanged with a 0.035-inch guidewire (Radifocus M; Terumo, Tokyo, Japan) through a transitional dilator. In the open approach, a small laparotomy was made by general surgeons in the right lower quadrant of the abdominal wall under general anesthesia. The terminal ileum was pulled out, and the serosa of the mesentery was incised to expose a peripheral vein of the superior mesenteric vein (SMV). After puncture with a 18-gauge needle, the distal side of the vein was ligated. Then, a 6 or 7 Fr sheath was placed in the peripheral PV or ileocolic vein. An angiographic catheter (5 Fr Kumpe; Cook, Bloomington, IN, USA / 4 Fr Hook; MEDIKIT, Tokyo, Japan) and 0.035-inch hydrophilic guidewire (Radifocus M; Terumo) were manipulated to pass the PV stenosis. A microcatheter (CXI support catheter; Cook / Veloute; ASAHI INTECC, Nagoya Japan / Tellus; ASAHI INTECC) and 0.014 or 0.016-inch microwire (Cruise; ASAHI INTECC / Meister; ASAHI INTECC) were also used as needed. Portography was obtained to evaluate the lesion length, degree of stenosis, vascular diameter around the stenosis, and collateral vein development. Self-expanding bare-metal stents (SMART Control; Cordis, Hialeah, FL, USA / Epic; Boston Scientific, Marlborough, MA, USA / E-Luminexx; Bard Medical / INNOVA; Boston Scientific) was placed to cover the stenotic lesion. Stent diameter was determined to be 1 to 2 mm larger than the healthy PV or SMV around the lesion. Stent length was selected to ensure coverage of the entire lesion (approximately 2 cm longer). In the case of severe stenosis or occlusion, pre-stent dilatation was performed using a 3 to 4 mm balloon catheter (Mustang; Boston Scientific). Post-stent dilatation was performed with a balloon catheter that was the same size or 1 mm smaller than the self-expandable stent. Post-stent portography was performed to evaluate PV inflow. At the end of the procedure with transhepatic approach, microfibrillar collagen (Avitene; Zeria Pharmaceutical Co., Tokyo, Japan) or 0.035-inch embolization coils (Tornado; Cook) were placed through the tract of liver parenchyma.

### Follow-up

After stent placement, anticoagulant therapy was administered unless abnormal coagulation profile or gastrointestinal bleeding. Heparin was administered for several days (5,000–10,000 units per day) and then one of the following oral anticoagulant agents: warfarin (dosed to maintain INR 1.5–2.0), edoxaban 30 or 60 mg/day, or apixaban 50 or 100 mg/day. The specific agent and its initiation were at the discretion of the physician. Stent patency was investigated using contrast-enhanced CT or US within one month of stent placement and then every three months or when symptoms of portal hypertension recurred.

### Definitions and data collection

The primary study endpoints were technical success, clinical success, and cumulative stent patency rate. The secondary endpoints were risk factors for stent occlusion. Technical success was defined as patent hepatopetal portal inflow with less than 50% residual stenosis and stent coverage of the entire stenotic lesion. Clinical success was defined according to clinical indications as follows: amelioration of gastrointestinal bleeding or refractory ascites within two weeks, amelioration of liver dysfunction within two weeks (transaminases < 45 units/L or serum ammonia < 80 µg/dL), prevention of gastrointestinal bleeding or refractory ascites, or initiation of chemotherapy. Stent occlusion was diagnosed on contrast-enhanced CT. The stent patency period was defined from the date of stent placement to the last date that stent patency was confirmed on CT. We chose 16 variables (described in Table 1) to examine association with stent occlusion by referring to previous studies of PV stent occlusion (Yamakado et al. 2001a; Lee et al. 2021; Kato et al. 2017; Tsuruga et al. 2013).

**Table 1:** Sixteen variables examined association with PV stent occlusion. (Location: After the sub-heading “Definitions and data collection” in the Materials and Methods)

1	Sex
2	Age at the time of PV stent placement (< 65 years old or ≥ 65 years old)
3	Underlying disease (pancreas cancer or other malignancy)
4	PV resection (performed or not)
5	Neoadjuvant radiotherapy (performed or not)
6	Etiology of PV stenosis (benign or malignant)
7	Degree of stenosis (stenosis or occlusion)
8	Lesion length (> 4 cm or ≤ 4 cm))
9	Interval between surgery and stent placement (≤ 100 days or > 100 days)
10	Approach (transhepatic or tran-ileocecal vein)
11	Hepatopetal collateral vein visualization before stent placement (absent or not)
12	Hepatofugal collateral vein visualization before stent placement (absent or not)
13	Hepatopetal collateral vein visualization after stent placement (absent or not)
14	Hepatofugal collateral vein visualization after stent placement (absent or not)
15	Post-procedural anticoaglants (absent or not)
16	Residual stenosis ≥ 30% after stent placement (absent or not)

*PV*, portal vein

Data were retrospectively collected by one of us in each institution. CT images before and after stent placement were reviewed to evaluate etiology of PV stenosis and stent patency. Malignancy-induced PV stenosis was diagnosed if a stenotic lesion was surrounded by a soft tissue density with any of the following characteristics: increase in size over time, worsening internal vascular irregularities on contrast-enhanced CT, or increased 18F-fluorodeoxyglucose uptake. PV occlusion was defined as a complete blockade of contrast medium on portography. Hepatofugal and hepatopetal collateral vein visualization were defined as retrograde flow through developed collateral veins, and intrahepatic PV flow through collateral veins around bile duct or hepaticojejunostomy anastomotic region, respectively. Collateral vein visualization was evaluated both before and after stent placement.

### Statistical analysis of risk factors for stent occlusion

A Cox proportional hazards model was used to determine risk factors of stent occlusion. Variables with *p*-value ≤ 0.1 in univariable analysis were included in the multivariable analysis. Outcomes are expressed as hazard ratios. Alpha was set at 0.05. Kaplan–Meier curves were constructed for variables showing *p*-value ≤ 0.05 in the multivariable analysis. Python version 3.8.5 (https://www.python.org/*)* and lifelines version 0.26.0 (https://lifelines.readthedocs.io/en/latest/) were used for the analysis.

## Results

### Short-term outcomes of PV stent placement

**Fig. 1:**
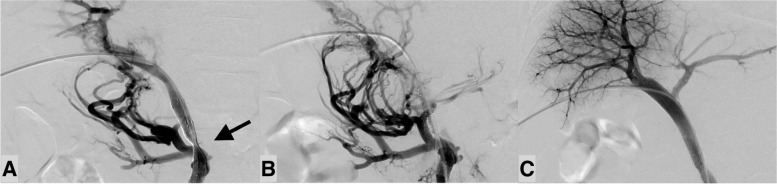
A patient with residual stenosis (in-stent) which resulted in stent occlusion. A man in his 70 s presented with hematochezia eight months after pancreaticoduodenectomy for pancreas cancer. Portal vein (PV) occlusion due to postoperative pancreatic fistula was confirmed on contrast-enhanced computed tomography (CT). In addition, development of hepatopetal collateral veins were observed around the choledochojejunostomy. Portography showed PV occlusion with hepatopetal collateral vein development. Two stents (SMART Control; Cordis, Hialeah, FL, USA) were placed through the occlusion and post-stent balloon dilatation was performed up to the nominal pressure. **A** Although residual in-stent stenosis (arrow) remained, PV flow improved at the end of the procedure. Contrast-enhanced CT was performed two days after stent placement and intra-stent thrombus was suspected. His hematochezia still persisted and anemia progressed, so additional treatment was scheduled. **B** Portography four days after the first stent placement shows recurrent PV occlusion. **C** After additional stent placement (SMART Control), hepatopetal PV flow had improved and flow through the collateral veins disappeared. The stent patency has been maintained for six years after the placement

**Fig. 2:**
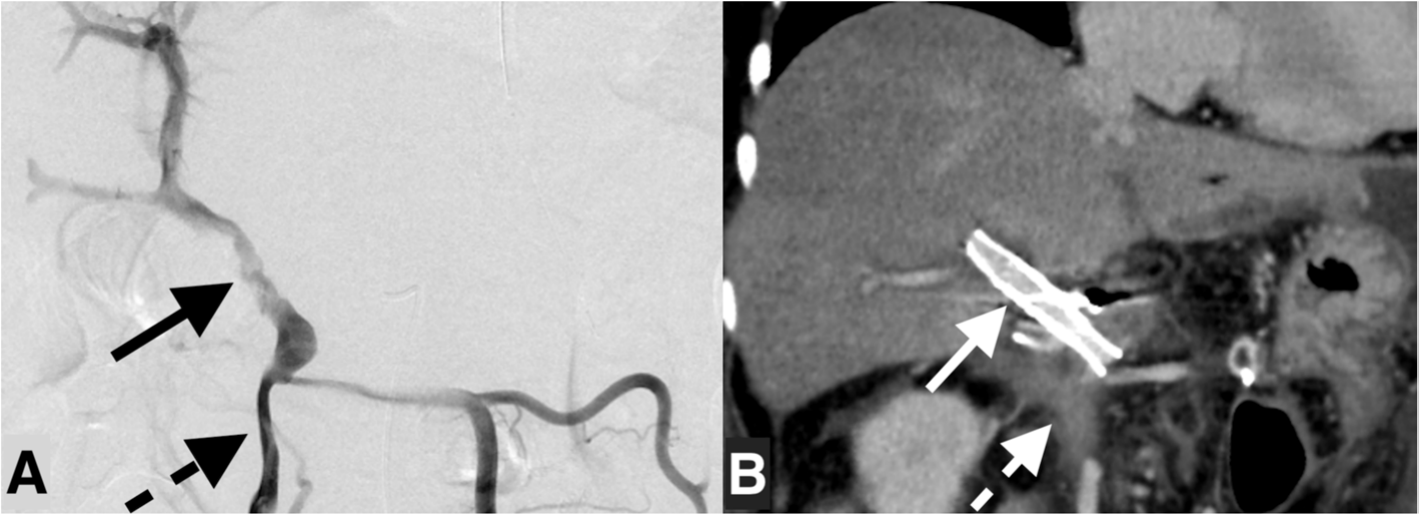
A patient with residual stenosis (outside-stent) which resulted in stent occlusion. A man in his 60 s presented with melena two months after subtotal stomach-preserving pancreatoduodenectomy for ampullary cancer. The contrast enhancement of the portal vein (PV) to superior mesenteric vein (SMV) was unclear and considered to be an occlusion or severe stenosis. Late-onset pancreatic juice leakage was considered as a cause. PV stent placement and total pancreatectomy (to control pancreatic juice leakage) was planned emergently. **A** Portography shows PV stenosis (arrow). Two stents (SMART Control; Cordis, Hialeah, FL, USA) were placed through the PV and post-stent balloon dilatation was performed up to the nominal pressure. Stent placement was not performed for SMV because it was unrecognized (dotted arrow). After the stent placement, PV flow improved. Subsequently, residual total pancreatectomy was performed. **B** Contrast-enhanced CT six days after the stent placement (arrow). The contrast enhancement of the SMV on the caudal side of the stent is unclear (dotted arrow). Two years and three months after placement, the stent occluded

Patient characteristics is described in Table 2. The indication of PV stent placement was to alleviate portal hypertension and/or to mitigate liver dysfunction in 29 patients, facilitation of chemotherapy in three, and prevention of gastrointestinal bleeding or refractory ascites in two. Venous sclerotherapy using 5% ethanolamine oleate was performed simultaneously with stent placement in one patient. Technical success was achieved in 30 patients (88.2%) and clinical success in 27 (79.4%). Four of 24 patients with portal hypertension-related symptoms and three of five patients with liver dysfunction failed to achieve clinical success. The four technical failures were as follows: unrecovered hepatopetal PV inflow through the stent (*n* = 1), residual stenosis > 50% (*n* = 3; Figs. 1 and 2). Rectus abdominis hematoma was seen in three patients who underwent open trans-ileocecal approach, but there were no other major complications related to the procedure.

**Table 2:** Patient characteristics (*n* = 34). (Location: After the sub-heading “Short-term outcomes of PV stent placement” in the Results)

	Number (%)
Sex (Male: Female)	15 (44.1%): 19 (55.9%)
Age^a^	67.0 ± 8.8
Underlying disease	
Pancreatic cancer	20 (58.8%)
Perihilar cholangiocarcinoma	7 (20.6%)
Decompensated liver cirrhosis	2 (5.9%)
Ampullary cancer	2 (5.9%)
Pancreatic cancer and Perihilar cholangiocarcinoma	1 (2.9%)
Hepatocellular carcinoma	1 (2.9%)
Gallbladder cancer	1 (2.9%)
Surgical procedures	
Subtotal stomach-preserving pancreatoduodenectomy	13 (38.2%)
Pancreatoduodenectomy	5 (14.7%)
Right lobectomy	3 (8.9%)
No surgery for pancreatic cancer	3 (8.9%)
Left trisectionectomy	3 (8.9%)
Liver transplantation	2 (5.9%)
Left lobectomy	2 (5.9%)
Hepatopancreatoduodenectomy	1 (2.9%)
Extended cholecystectomy	1 (2.9%)
Distal pancreatectomy	1 (2.9%)
Symptoms	
Ascites	9 (26.5%)
Liver dysfunction	5 (14.7%)
Gastrointestinal bleeding	5 (14.7%)
Asymptomatic (for introducing chemotherapy or preventing portal hypertension-related symptoms)	5 (14.7%)
Gastrointestinal bleeding, ascites	2 (5.9%)
Encephalopathy	2 (5.9%)
Ascites, diarrhea	2 (5.9%)
Liver dysfunction, ascites	1 (2.9%)
Encephalopathy, ascites	1 (2.9%)
Diarrhea	1 (2.9%)
Intraperitoneal bleeding	1 (2.9%)
Range of PV stenosis or occlusion	
PV to SMV	16 (47.1%)
PV	12 (35.3%)
SMV	5 (14.7%)
PV resection	14 (41.2%)
Neoadjuvant radiotherapy	13 (38.2%)
Etiology of PV stenosis (benign vs malignant)	20 (58.8%): 14 (41.2%)
Degree of stenosis (stenosis vs occlusion)	16 (47.1%): 18 (52.9%)
Lesion length (mm)^a^	41.5 ± 16.0
Stent diameter (mm)^a^	8.8 ± 1.5
Interval days between surgery and stent placement^b^	101 (32–448)
Approach (transhepatic vs trans-ileocecal vein)	18 (52.9%): 16 (47.1%)
Collateral vein (hepatofugal, hepatopetal)	5 (14.7%): 7 (20.6%)
Residual stenosis ≥ 30% after stent placement (in-stent, outside-stent)	3 (8.9%): 1 (2.9%)
Post-procedural anticoagulants	
Warfarin	16 (47.1%)
Edoxaban (Lixiana®)	10 (29.4%)
None	6 (17.6%)
Apixaban (Eliquis®)	2 (5.9%)
Stent occlusions	6 (17.6%)

Data are the number of patients, with percentage in parentheses

^a^Data are mean ± standard deviation

^b^Data are median, with interquartile range in parentheses

*PV* portal vein, *SMV* superior mesenteric vein

### Long-term outcomes of PV stent patency

During the median CT follow-up of 175.5 days (interquartile range, 26–572), six stent occluded due to thrombosis (*n* = 5) and tumor ingrowth (*n* = 1). The cumulative stent patency rate at six months, one year, and three years was 79.1%, 79.1%, and 65.9%, respectively. Among the six occlusions, one was treated with additional balloon dilatation and coil embolization of collateral vein (developed left gastric vein), and another with additional stent placement. The remaining four were treated with heparinization and anticoagulant therapy; among these, only two patients who underwent additional interventional procedures acquired stent patency.

### Statistical analysis of risk factors for stent occlusion

**Fig. 3:**
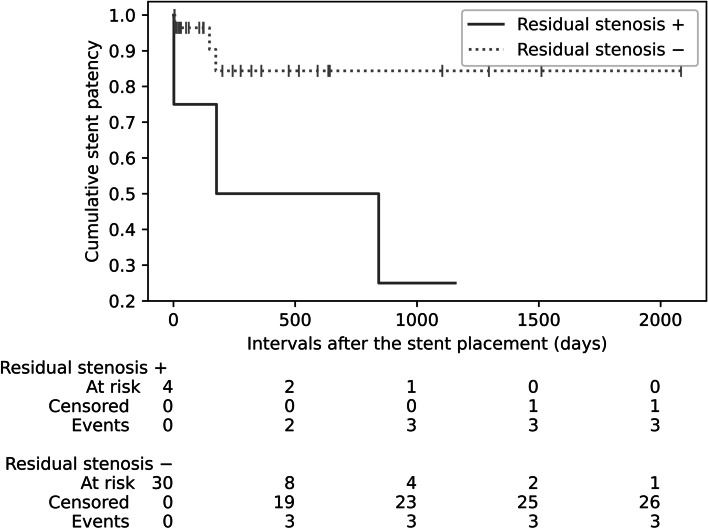
Comparison of cumulative stent patency in patients with and without residual stenosis after stent placement. The black plot shows the cumulative stent patency rate in patients with residual stenosis after stent placement. The gray dotted plot shows the cumulative stent patency rate in patients without residual stenosis after stent placement. The vertical lines on the gray plot represent censoring. The figures at the bottom count the cumulative number of patients at risk, censored, and with stent occlusion

In the univariate analysis, the variables with a *p*-value ≤ 0.1 were lesion length over 4 cm, hepatofugal collateral vein visualization after stent placement, and residual stenosis > 30% after stent placement. The multivariate analysis using these three variables showed that residual stenosis > 30% after stent placement was significantly associated with stent occlusion (hazard ratio, 10.80; 95% confidence interval, 1.08–108.44; *p* = 0.04) (Table 3). Figure 3 shows the Kaplan–Meier curves of the patients with and without residual stenosis > 30% after stent placement.

**Table 3:** Univariable and multivariable Cox regression analysis of risk factors for stent occlusion. (Location: After the sub-heading “Statistical analysis of risk factors for stent occlusion” in the Results)

Variable	Univariable analysis		Multivariable analysis	
	Hazard ratio*	*p* value	Hazard ratio*	*p* value
Sex				
female	1 [reference]			
male	0.69 (0.12, 3.88)	0.67		
Age				
< 65 years old	1 [reference]			
≥ 65 years old	0.61 (0.12, 3.06)	0.55		
Underlying disease				
other malignancy	1 [reference]			
pancreas cancer	0.60 (0.12, 3.03)	0.54		
PV resection	0.24 (0.03, 2.04)	0.19		
Neoadjuvant radiotherapy	0.73 (0.13, 4.02)	0.72		
Etiology of PV stenosis				
benign	1 [reference]			
malignant	0.43 (0.05, 3.87)	0.45		
Degree of stenosis				
stenosis	1 [reference]			
occlusion	2.15 (0.39, 11.95)	0.38		
Lesion length				
≤ 4 cm	1 [reference]			
> 4 cm	10.16 (1.14, 88.63)	0.04	5.30 (0.53, 53.18)	0.15
Interval between surgery and stent placement				
≤ 100 days	1 [reference]			
> 100 days	3.29 (0.57, 19.15)	0.18		
Approach				
transhepatic	1 [reference]			
trans-ileocecal vein	0.82 (0.16, 4.14)	0.81		
Hepatopetal collateral vein visualization before stent placement	2.06 (0.38, 11.26)	0.41		
Hepatofugal collateral vein visualization before stent placement	3.00 (0.55, 16.48)	0.21		
Hepatopetal collateral vein visualization after stent placement	1.14 (0.00, 63,019.87)	0.98		
Hepatofugal collateral vein visualization after stent placement	7.76 (1.27, 47.24)	0.03	8.58 (0.66, 112.18)	0.10
Post-procedural anticoaglants	8.1 × 10^6^ (0.00, infinite)	1.00		
Residual stenosis ≥ 30% after stent placement	5.13 (0.99, 26.54)	0.05	10.80 (1.08, 108.44)	0.04

*The 95% confidence interval for each point estimate is shown in parentheses

## Discussion

This study examined outcomes of PV stent placement and risk factors for stent occlusion. PV stent placement was technically successful in 88.2% of patients and most (79.4%) experienced considerable improvement of portal hypertension or liver dysfunction. The cumulative stent patency rate at six months, one year, and three years was 79.1%, 79.1%, and 65.9%, respectively. Six of 34 patients experienced stent occlusion. Residual stenosis > 30% after stent placement was the only significant factor associated with stent occlusion.

The efficacy of PV stent has been shown in previous studies, especially for stenosis at the PV anastomosis site after liver transplantation (Kim et al. 2016, 2019; Narita et al. 2019). The five-year patency rate after liver transplantation is greater than 80% (Shim et al. 2018; Wei et al. 2009). Several recent studies have investigated PV stent placement after hepatobiliary-pancreatic surgery (HBPS), and the stent patency rate was reported as 74.8% at 1 year (Lee et al. 2021) and 74.6% at the mean observation period of 13 months (Zhou et al. 2014). In this study, most of the patients (85.3%) underwent PV stent placement after HBPS and the one-year cumulative stent patency rate of 79.1% is comparable to the previous studies. These patency results seemed acceptable, but worse than after liver transplantation, probably due to technical problems with wire crossing or tumor recurrence. PV stenosis lesions after HBPS tend to be lengthy (Kato et al. 2017) and successful guidewire crossing tends to be challenging (failure rate, 5%–14%) (Shim et al. 2017; Kim et al. 2011; Hyun et al. 2017), especially in patients who undergo PV resection or radiation therapy (Hyun et al. 2017). Though technical failure occurred in four patients, guidewire crossing was successful in all patients in this study. The ileocecal approach was selected in a relatively large proportion of patients (47.1%), which may be a reason why guidewire crossing was successful in all. The trans-ileocecal vein approach enables easier advancement of the guidewire through the thin portal branches (Sawai et al. 2019) and portography from the side peripheral to the stenosis allows understanding of the details of the stenotic lesions.

Four patients had residual stenosis > 30% after stent placement (three in-stent stenosis and one outside-stent stenosis) and three developed stent occlusions. In coronary and carotid stents, residual stenosis has been reported as a risk factor for stent occlusion by stagnated blood flow (Fujii et al. 2005; Tao et al. 2020). It seems reasonable that the same applies to PV stents. In the patients with residual in-stent stenosis, sufficient stent dilation was not possible because of severe pain in two and a hard lesion which could not be released by the balloon catheter in one. Although efforts are needed to mitigate in-stent stenosis, excessive balloon dilatation might cause PV injury (Thompson et al. 2020; Piardi et al. 2016). Figure 1 shows a patient with in-stent stenosis who ended up with stent occlusion. Although balloon dilation beyond the nominal pressure was not performed at the procedure, it might be better to release the stenosis until hepatopetal collateral vein visualization disappears. In the patient with outside-stent stenosis, the residual stenotic lesion in the SMV was unrecognized during the procedure because it was different from the most narrowed area of the PV (Fig. 2). In addition to the low SMV perfusion, the splenic vein had been surgically resected in this patient. Severely decreased PV inflow could be the cause of stent occlusion and additional SMV stent might have mitigated the risk. Careful evaluation of the lesion’s range on preoperative CT is essential to avoid missing stenotic lesions. Referring to a landmark such as the splenic vein or inferior mesenteric vein would be helpful. A previous study (Inui et al. 2019) showed a technique with intravascular ultrasound (IVUS) for safety PV recanalization for chronic PV occlusion. We assume that IVUS would also help to assess the range of stenosis correctly.

Though there was no significant difference (*p* = 0.10), it was notable that three of the four patients with residual collateral vein visualization after stent placement experienced stent occlusions. A previous study showed that developed collateral vein was significantly associated with stent occlusion; the investigators recommended collateral vein embolization when observed (Kato et al. 2017). Another study of PV stent placement for jejunal variceal bleeding reported that both hemostasis and stent patency could not be obtained by PV stent placement alone; additional variceal embolization was necessary to achieve hemostasis and stent patency (Shim et al. 2017). Our study might lack sufficient statistical power because only four patients had visualized collateral vein after stent placement. Embolization of collateral veins might be effective to maintain stent patency by increasing stent inflow; however, embolization of collateral veins may lead to refractory portal hypertension or liver dysfunction. It remains unclear whether PV stent placement alone is sufficient to relieve portal hypertension (Shim et al. 2017). If collateral vein visualization remains after stent placement, embolization might be preferable after considering liver function and the degree of collateral vein development. Further studies are warranted to determine indications for collateral vein embolization.

We used anticoagulant therapy unless there was an abnormal coagulation profile or gastrointestinal bleeding. A previous study (Kato et al. 2017) showed that anticoagulant therapy was significantly associated with PV stent patency. In addition, some other studies used anticoagulation therapy after PV stent placement (Hasegawa et al. 2015; Yamakado et al. 2001b; Zhou et al. 2014). However, to the best of our knowledge, no evidence exists about the superiority of anticoagulant therapy over antiplatelet agents. General drug usage after PV stent placement needs to be established.

Our study had several limitations. First, it was retrospective in design and the subjects and procedures were heterogeneous; therefore, selection bias may have been introduced. Second, the sample size was small, and the effect of collateral vein embolization could not be considered. Third, we did not examine pressure gradient measurements, which can be used in stent placement decision making.

In summary, PV stent placement was technically feasible and effective in improving portal hypertension-related symptoms or liver dysfunction. Stent occlusion was not uncommon and residual stenosis > 30% after stent placement may be the risk factor. We should pay attention to assess the range of stenosis correctly and release the stenosis as much as possible.

## Data Availability

The datasets used and analyzed during the current study are available from the corresponding author on reasonable request.

## Abbreviations

PV: Portal vein
SMV: Superior mesenteric vein
HBPS: Hepatobiliary-pancreatic surgery
IVUS: Intravascular ultrasound

## References

[CR1] Fujii K, Carlier SG, Mintz GS, et al (2005) Stent underexpansion and residual reference segment stenosis are related to stent thrombosis after sirolimus-eluting stent implantation. J Am College Cardiol. 995–998. 10.1016/j.jacc.2004.12.066 10.1016/j.jacc.2004.12.066 15808753

[CR2] Hasegawa T, Yamakado K, Takaki H (2015). Portal Venous Stent Placement for Malignant Portal Venous Stenosis or Occlusion: Who Benefits?. Cardiovasc Intervent Radiol.

[CR3] Hiyoshi M, Fujii Y, Kondo K, Imamura N, Nagano M, Ohuchida J (2015). Stent placement for portal vein stenosis after pancreaticoduodenectomy. World J Surg..

[CR4] Hyun D, Park KB, Cho SK (2017). Portal Vein Stenting for Delayed Jejunal Varix Bleeding Associated with Portal Venous Occlusion after Hepatobiliary and Pancreatic Surgery. Korean J Radiol.

[CR5] Inui S, Kondo H, Yamamoto M (2019). Intravascular ultrasound-guided percutaneous portal vein recanalization for chronic portal vein obstruction. J Vasc Interv Radiol.

[CR6] Kato A, Shimizu H, Ohtsuka M, Yoshitomi H, Furukawa K, Miyazaki M (2017). Portal vein stent placement for the treatment of postoperative portal vein stenosis: long-term success and factor associated with stent failure. BMC Surg.

[CR7] Kim KR, Ko G-Y, Sung K-B, Yoon H-K (2011). Percutaneous transhepatic stent placement in the management of portal venous stenosis after curative surgery for pancreatic and biliary neoplasms. AJR Am J Roentgenol.

[CR8] Kim KS, Kim JM, Lee JS, Choi GS, Cho J-W, Lee S-K (2019). Stent insertion and balloon angioplasty for portal vein stenosis after liver transplantation: long-term follow-up results. Diagn Interv Radiol.

[CR9] Kim S, Jung H, Kim J, Yim N, Kim H (2016) Long-term outcomes of percutaneous transhepatic balloon angioplasty with stent deployment for portal vein stenosis after liver transplantation. J Vasc Interv Radiol. S282. 10.1016/j.jvir.2015.12.716

[CR10] Kumar A, Sharma P, Arora A (2015). Review article: portal vein obstruction–epidemiology, pathogenesis, natural history, prognosis and treatment Aliment Pharmacol Ther. Wiley.

[CR11] Lee JH, Yoon CJ, Choi WS (2021). Transhepatic stent placement for portal vein obstruction after hepatobiliary and pancreatic surgery: long-term efficacy and risk factor for stent failure. Eur Radiol.

[CR12] Narita Y, Sugawara Y, Ibuki S (2019). Portal vein stent placement in living-donor liver transplantation: a single-center experience. Transplant Proc.

[CR13] Piardi T, Lhuaire M, Bruno O (2016). Vascular complications following liver transplantation: A literature review of advances in 2015. World J Hepatol.

[CR14] Sawai Y, Kokudo T, Sakamoto Y (2019). Stent placement for benign portal vein stenosis following pancreaticoduodenectomy in a hybrid operating room. Biosci Trends.

[CR15] Settmacher U, Nüssler NC, Glanemann M (2000). Venous complications after orthotopic liver transplantation. Clin Transplant.

[CR16] Shim DJ, Ko G-Y, Sung K-B, Gwon DI, Ko HK (2018). Long-term outcome of portal vein stent placement in pediatric liver transplant recipients: a comparison with balloon angioplasty. J Vasc Interv Radiol.

[CR17] Shim DJ, Shin JH, Ko G-Y (2017). Portal vein stent placement with or without varix embolization of jejunal variceal bleeding after hepatopancreatobiliary surgery. Acta Radiol.

[CR18] Tao Y, Hua Y, Jia L, Jiao L, Liu B (2020). Risk factors for residual stenosis after carotid artery stenting. Front Neurol.

[CR19] Thompson SM, Fleming CJ, Yohanathan L, Truty MJ, Kendrick ML, Andrews JC (2020). Portomesenteric venous complications after pancreatic surgery with venous reconstruction: imaging and intervention. Radiographics.

[CR20] Tsuruga Y, Kamachi H, Wakayama K (2013). Portal vein stenosis after pancreatectomy following neoadjuvant chemoradiation therapy for pancreatic cancer. World J Gastroenterol.

[CR21] Wei B-J, Zhai R-Y, Wang J-F, Dai D-K, Yu P (2009). Percutaneous portal venoplasty and stenting for anastomotic stenosis after liver transplantation. World J Gastroenterol.

[CR22] Woo DH, Laberge JM, Gordon RL, Wilson MW, Kerlan RK (2007). Management of portal venous complications after liver transplantation. Tech Vasc Interv Radiol.

[CR23] Yamakado K, Nakatsuka A, Tanaka N, Fujii A, Terada N, Takeda K (2001). Malignant portal venous obstructions treated by stent placement: significant factors affecting patency. J Vasc Interv Radiol.

[CR24] Yamakado K, Nakatsuka A, Tanaka N (2001). Portal venous stent placement in patients with pancreatic and biliary neoplasms invading portal veins and causing portal hypertension: initial experience. Radiology.

[CR25] Zhou ZQ, Lee JH, Song KB (2014). Clinical usefulness of portal venous stent in hepatobiliary pancreatic cancers. ANZ J Surg.

